# Assessment of unvaccinated and vaccinated patients with coronavirus disease 2019 (COVID-19) treated with monoclonal antibodies during the delta wave (July 1–August 20, 2021): a retrospective observational monocentric study

**DOI:** 10.1186/s12879-022-07626-6

**Published:** 2022-07-27

**Authors:** Yi Guo, Kelsie Cowman, Mei Chang, Hongkai Bao, Austin Golia, Terrence Mcsweeney, Linda Bard, Roxanne Simpson, Erin Andrews, Liise-anne Pirofski, Priya Nori

**Affiliations:** 1 Department of Pharmacy, Montefiore Medical Center Moses, 111 East 210th Street, Bronx, NY 10467 USA; 2 Division of Infectious Diseases, Department of Medicine, Montefiore Medical Center, Albert Einstein College of Medicine, 3411 Wayne Avenue, #4H, Bronx, NY 10467 USA; 3 Network Performance Group, Montefiore Health System, Bronx, NY USA; 4 Department of Pharmacy, Montefiore Medical Center Weiler, 1825 Eastchester Rd, Bronx, NY 10461 USA; 5 Department of Pharmacy, Montefiore Medical Center Wakefield, 500 East 233rd St, Bronx, NY 10466 USA; 6 Faculty Practice Group, Montefiore Medical Center, 111 East 210th Street, Bronx, NY 10467 USA; 7 Department of Nursing, Montefiore Medical Center, 111 East 210th Street, Bronx, NY 10467 USA; 8 Network Performance Group, Montefiore Health System, 5 Executive Plaza, Suite 112B, Yonkers, NY 10701 USA; 9 Division of Infectious Diseases, Department of Microbiology and Immunology, Department of Medicine, Montefiore Medical Center, Albert Einstein College of Medicine, 1300 Morris Park Ave, Bronx, NY 10461 USA

**Keywords:** Coronavirus disease 19, COVID-19, Delta variant, SARS-CoV-2 variant B.1.617.2, Monoclonal antibody, Casirivimab/imdevimab, Vaccination, Vaccinated, Unvaccinated

## Abstract

**Background:**

Monoclonal antibodies (mAb) prevent COVID-19 progression when administered early. We compared mAb treatment outcomes among vaccinated and unvaccinated patients during Delta wave and assessed the feasibility of implementing stricter eligibility criteria in the event of mAb scarcity.

**Methods:**

We conducted a retrospective observational study of casirivimab/imdevimab recipients with mild-to-moderate COVID-19 infection in an emergency department or outpatient infusion center (July 1–August 20, 2021). Primary outcome was all-cause hospital admission within 30 days post-treatment between vaccinated vs. unvaccinated patients during Delta surge in the Bronx, NY.

**Results:**

A total of 250 patients received casirivimab/imdevimab (162 unvaccinated vs. 88 vaccinated). The median age was 39 years for unvaccinated patients, and 52 years for vaccinated patients (p < 0.0001). The median number of EUA criteria met was 1 for unvaccinated and 2 for vaccinated patients (p < 0.0001). Overall, 6% (15/250) of patients were admitted within 30 days post-treatment. Eleven unvaccinated patients (7%) were admitted within 30-days compared to 4 (5%) vaccinated patients (p = 0.48).

**Conclusions:**

All-cause 30-day admission was not statistically different between vaccinated and unvaccinated patients. When federal allocation of therapies is limited, programs must prioritize patients at highest risk of hospitalization and death regardless of vaccination status.

## Background

Multiple studies demonstrate the efficacy of monoclonal antibodies (mAb) in preventing progression of COVID-19 when administered to high-risk patients early in disease presentation [1–8]. A study by Cooper and colleagues evaluated nearly 3000 patients with coronavirus disease 2019 (COVID-19) who received mAb therapies in Houston, TX [9]. Compared to a propensity-matched control cohort, mAb-treated patients had significantly lower rates of hospitalization at 14- and 28-days and decreased mortality at 28-days post-infusion [9]. However, the impact of circulating SARS-CoV-2 variants was not addressed. A study conducted at our medical center in the Bronx, NY revealed a concerning increase in bamlanivimab treatment failures between January and April 2021, likely due to an increase in the locally circulating variant B.1.526, which was resistant to bamlanivimab monotherapy [10]. Casirivimab/imdevimab remained active against B.1.526 [11]. To date, there is a paucity of data addressing regional heterogeneity, outcomes during the Delta surge, or vaccination status of mAb-treated COVID-19 patients. The SARS-CoV-2 variant B.1.617.2 was identified in 70–98% of sequenced isolates across multiple New York City zip codes until July 2021 [12]. Despite the high efficacy of mRNA vaccines against severe disease, hospitalization, and death, an increasing number of breakthrough SARS-CoV-2 infections due to the Delta variant were reported [13, 14], potentially due to waning antibody levels [15]. The Bronx has had lower vaccine uptake among New York City boroughs. Its residents remain particularly vulnerable to SARS-CoV-2 infection [16] and increasing access to COVID-19 mAb therapies is a priority here.

In December 2020, our antimicrobial stewardship team (AST) established a COVID-19 infusion program to treat high-risk patients in the Bronx as per Food and Drug Administration (FDA) emergency use authorization (EUA) criteria [10]. In May 2021, the FDA expanded treatment criteria by lowering the body mass index (BMI) threshold from 35 to 25 kg/m^2^ and including additional comorbidities [17, 18]. Additional medical comorbidities or demographics (e.g., race or ethnicity) may also increase an individual patients’ risk for progression to severe COVID-19 [19]. Importantly, patients with history of COVID-19 vaccination are not excluded from mAb eligibility criteria. In June 2021, FDA authorized lowering the dose to 1200 mg (600 mg of casirivimab and 600 mg of imdevimab), which is half the dose originally authorized. Our patients were treated based on the expanded criteria and updated dosing as per FDA EUA criteria.

In September 2021, the US Health and Human Services Administration modified the mAb allocation process whereby the quantity of doses received by each state was proportional to COVID-19 incidence [20]. The new federal strategy impacted workflows of programs serving communities with high SARS-CoV-2 transmission and lower vaccine uptake within states with lower relative COVID-19 burden, such as NYS during the Delta surge [20].

The primary objective of this study was to evaluate mAb treatment outcomes among vaccinated and unvaccinated patients during the Delta surge in the Bronx, NY. The secondary objective was to assess the feasibility of implementing stricter mAb eligibility criteria in the event of resource scarcity in New York City.

## Methods

**Fig. 1:**
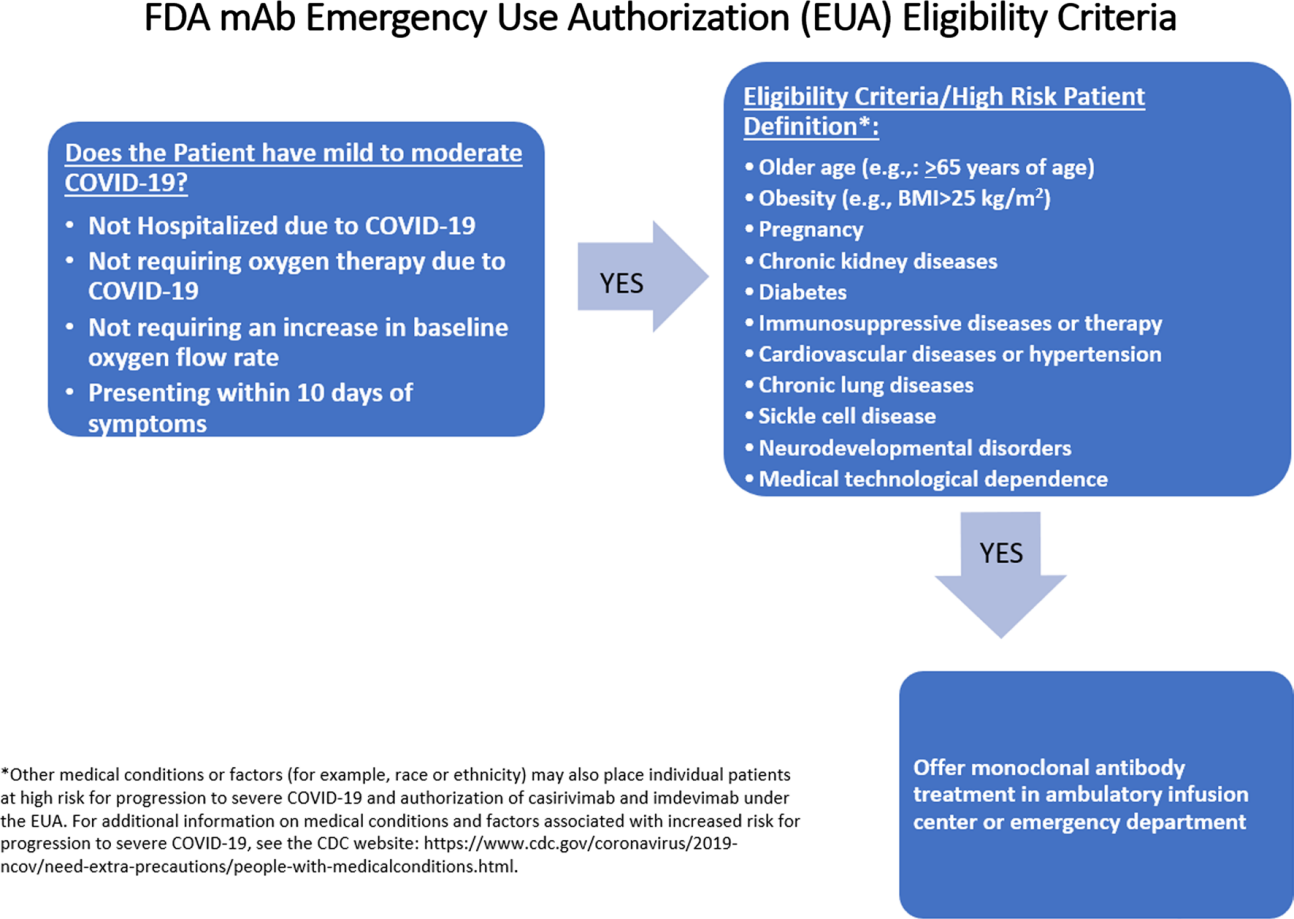
FDA Emergency Use Authorization (EUA) criteria of casirivimab/imdevimab

We conducted a retrospective observational study of patients with mild-to-moderate COVID-19 meeting expanded EUA criteria received casirivimab/imdevimab in an emergency department (ED) or outpatient infusion center at Montefiore Medical Center between July 1 and August 20, 2021 [17, 18]. The FDA EUA inclusion/exclusion criteria for casirivimab/imdevimab are summarized in Fig. 1.

Our primary outcome was all-cause hospital admission 30 days post-treatment. COVID-19-related admissions were defined as known symptoms or sequelae (e.g., worsening respiratory status or thromboembolic complications), as determined by the study team [10]. Using the National Institutes of Health COVID-19 treatment guidelines [21]. Mild illness was defined as usual signs and symptoms of COVID-19 (e.g., fever, cough, sore throat, malaise, headache, muscle pain, nausea, vomiting, diarrhea, loss of taste and smell) without shortness of breath, dyspnea on exertion, or abnormal imaging. Moderate illness is defined as evidence of lower respiratory disease with SpO_2_ ≥ 94% on room air at sea level. Severe illness is defined as a SpO_2_ < 94% on room air at sea level, PaO_2_/FiO_2_ < 300 mm Hg, a respiratory rate > 30 breaths/min, or infiltrates in > 50% of lung fields. Critical illness is defined as acute respiratory distress syndrome, septic shock representing virus-induced distributive shock, cardiac dysfunction, an exaggerated inflammatory response, and/or exacerbation of underlying comorbidities. Patients were considered fully vaccinated two weeks following receipt of 2 doses of BNT162b2 or mRNA-1273, or 1 dose of Ad26.COV2.S. Immunocompromise was defined as having HIV, liver cirrhosis, sickle cell anemia, asplenia, malignancy, solid organ transplant, bone marrow transplant, active chemotherapy treatment within the past 30 days, chronic use of steroids, tacrolimus, mycophenolate, or biologics [10]. Institutional prioritization schema utilized September 2021 onward is shown in Table 1.

**Table 1:** Institutional prioritization schema for monoclonal antibody therapy for patients meeting EUA criteria during the delta wave

	Preferred option	Alternative option
Unvaccinated/partially vaccinated AND symptomatic ≤ 7 days^a^	Immediate treatment (Emergency Department or same day in infusion center)	Next day scheduling at infusion center
Fully vaccinated > 4 or more months ago OR suspected poor response to vaccine, AND symptomatic ≤ 7 days^a^	Immediate treatment (Emergency Department or same day in infusion center)	Next day referral at infusion center
Unvaccinated/partially vaccinated AND asymptomatic	Wait and watch, reassess for treatment in 48 h if symptom progression	
Fully vaccinated AND asymptomatic	Hold off on treatment	
Fully vaccinated AND mildly symptomatic ≤ 7 days*	Wait and watch, reassess for treatment in 48 h if symptom progression	
Post-exposure prophylaxis (PEP) (recent significant exposure in high-risk unvaccinated patient OR severely immunocompromised patient without recent vaccination or no documented response by serologic testing)	If sufficient doses available for PEP, treat in ED or other non-COVID location as soon as possible; requires same-day negative SARS-CoV-2 PCR)	

^a^Patients with immune compromise, suspected poor response to vaccines, and potential prolonged viral shedding can be treated up to 10 days from symptom onset per Emergency Use Authorization criteria; use SARS-CoV-2 PCR cycle threshold (Ct) value (if available) to help guide clinical judgement; ONLY use Ct value in clinical context

Patients admitted due to COVID-19 progression on the same day as mAb treatment were excluded.^8^ Bivariate analyses were conducted using X^2^, Fisher’s exact test, or t test, as appropriate. Multivariable analysis was conducted using logistic regression. All statistical tests were 2-tailed and p < 0.05 were considered significant. Analyses were conducted using SAS, version 9.4 software (SAS Institute, Cary, NC). The Albert Einstein College of Medicine institutional review board approved the study with waiver of informed consent (IRB# 2020-12525).

## Results

Between July 1 and August 20, 2021, 250 COVID-19 patients received casirivimab/imdevimab; 162 (65%) were unvaccinated and 88 (35%) were vaccinated at time of treatment. The median age was 39 years for unvaccinated patients, and 52 years for vaccinated patients (p < 0.0001). Overall, 101 (40%) of patients were Hispanic, 89 (36%) non-Hispanic Black, and 31 (12%) non-Hispanic white. Median BMI was not significantly different between the unvaccinated (30) and vaccinated (30) groups (p = 0.87). Vaccinated patients had shorter duration of COVID-19 symptoms prior to treatment (median 3 vs. 4 days, p = 0.02). The most common risk factor was BMI > 25 kg/m^2^, observed in 115 (71%) unvaccinated and 67 (76%) vaccinated patients (p = 0.66). The median number of EUA criteria met was 1 for unvaccinated and 2 for vaccinated patients (p < 0.0001) (Table 2). Cycle threshold (Ct) values were available for 104 (64%) of unvaccinated and 36 (37%) of vaccinated patients, with no significant differences between groups (Table 2).

**Table 2:** Characteristics of SARS-CoV-2-positive patients treated with monoclonal antibodies by vaccine status prior to treatment

	Non-vaccinated n (%) n = 162	Vaccinated n (%) n = 88	p-value
Age, median (IQR)	39 (29–47)	52 (41–63)	< 0.0001
Sex			0.65
Male	73 (45)	37 (42)	
Female	89 (55)	51 (58)	
Race/ethnicity			0.0002
Hispanic	68 (42)	33 (38)	
Non-Hispanic Black	69 (43)	20 (23)	
Non-Hispanic White	11 (7)	20 (23)	
Asian	0 (0)	1 (1)	
Other	6 (4)	5 (6)	
Unavailable	8 (5)	9 (10)	
BMI	30 (25–35)	30 (26–35)	0.87
High-risk co-morbidities per EUA*			
BMI ≥ 25 kg/m^2^	115 (71)	67 (76)	0.66
Pregnancy	7 (4)	0 (0)	0.054
Chronic lung disease	33 (20)	18 (20)	0.99
Chronic kidney disease	1 (1)	5 (6)	0.0045
Diabetes mellitus	22 (14)	25 (28)	0.004
Immunocompromised conditions**	12 (7)	13 (15)	0.06
Medical-related technological dependence	1 (1)	2 (2)	0.25
Neurodevelopmental disorders	5 (3)	0 (0)	0.17
Cardiovascular disease or hypertension	28 (17)	36 (41)	< 0.0001
Number of EUA criteria met	1 (1–2)	2 (1–3)	< 0.0001
Symptom Duration Prior to Treatment			
Days, median (IQR)	4 (3–6)	3 (2–5)	0.02
Asymptomatic, n (%)	0	0	
Exact days unavailable, n (%)	0	1 (1)	
Cycle threshold (Ct) values	n = 104	n = 36	0.06
< 25	70 (67)	25 (69)	
Between 25 and 35	30 (29)	6 (17)	
> 35	4 (4)	5 (14)	
Days between vaccination and treatment, median (IQR)			
Admitted (all-cause 30-day admission)	–	154 (118–166)	
Not admitted	–	160 (133–190)	

Hospitalization	n = 11	n = 4	
Outcomes			
All-cause 30-day admission	11 (7)	4 (5)	0.48
COVID-19-related 30-day admission	7 (4)	1 (1)	0.27
Presenting symptoms at admission			
Shortness of breath	7 (64)	1 (25)	
Cough	3 (27)	1 (25)	
Fatigue	4 (36)	0	
Chest X-ray upon admission	9 (82)	3 (75)	
Requiring supplemental oxygen	3 (27)	0	
Asymptomatic	4 (36)	3 (75)	
Mild illness	1 (9)	0	
Moderate illness	6 (55)	1 (25)	
Severe or critically illness	0	0	
Lab values on admission (where performed)			
White blood cell^†^ (K/uL), median (IQR)	7.5 (6–8.9)	8.7 (6–12.6)	
C-reactive protein^††^ (mg/dL), median (IQR)	4.5 (3.2–9.3)	11.8 (9.5–14.2)	
D-dimer^§^ (ug/mL), median (IQR)	0.56 (0.42–1.2)	0.66	
Fibrinogen^§§^ (ug/mL), median (IQR)	456 (384.8–591.8)	585	
Lactic dehydrogenase^¶^ (U/L), median (IQR)	284 (232.3–344.8)	202.5 (198.3–206.8)	

*EUA: emergency use authorization

**Immunocompromised conditions: HIV, cirrhosis of the liver, sickle cell anemia, asplenia, malignancy, solid organ transplant, bone marrow transplant, patient received chemotherapy, chronic steroids, tacrolimus, mycophenolate, biologics, etc.

^†^15 patients had white blood cell test values available

^††^8 patients had C-reactive protein value available

^§^8 patients had D-dimer value available

^§§^7 patients had fibrinogen value available

^¶^10 patients had lactic dehydrogenase value available

30-day

Overall, 6% (15/250) of patients were admitted to our hospital within 30 days of mAb treatment. Eleven unvaccinated patients (7%) were admitted (all-cause) within 30-days compared to 4 (5%) of vaccinated patients (p = 0.48). One (1%) vaccinated patient had a COVID-19-related admission compared to 7 (4%) unvaccinated patients (p = 0.27). When adjusting for age and number of EUA risk factors, vaccine status was still not significantly associated with either all-cause (OR 0.55, CI 0.14–1.83, p = 0.30) or COVID-related 30- day admission (OR 0.19, CI 0.01–1.71, p = 0.13) (Table 3). The non-COVID-19 reasons for admission were childbirth, oligohydramnios, lower extremity pain without deep vein thrombosis, sickle cell crisis, intra-abdominal pain, elective surgery, and abscess. Shortness of breath, cough, and fatigue were the most common symptoms reported by patients upon admission for COVID-19 progression. Chest X-rays were obtained for 80% (12/15) patients. Three patients did not receive a chest X-ray due to asymptomatic status and admission for non-COVID-19 reasons. Among the 15 admitted patients, 7 patients were asymptomatic, 1 had mild illness, 7 had moderate illness, and none had severe or critical illness requiring intubation. For patients with labs available upon admission, the median white blood cell was 7.5 K/μL (n = 15), C-reactive protein 6.2 mg/dL (n = 8), D-dimer 0.61 μg/mL (n = 8), fibrinogen 501 μg/mL (n = 7), and lactic dehydrogenase 250 U/L (n = 10). Median duration between vaccination and mAb treatment was 160.5 days; 153.5 days for admitted and 160.5 days for non-admitted patients.

**Table 3:** Multivariable analysis of vaccine status and 30-day hospital admission, adjusting for age and number of EUA risk factors

Outcome	Adjusted odds ratio (95% confidence interval)	p-value
All-cause 30-day hospital admission	0.55 (0.14–1.83)	0.30
COVID-19 related 30-day hospital admission	0.19 (0.01–1.71)	0.13

## Discussion

Although low absolute admission numbers, mAb recipients had a similar all-cause 30-day admissions regardless of vaccination status supporting FDA in-vitro data indicating retained activity of casirivimab/imdevimab against B.1.617.2 [17]. Among the 250 treated patients, the majority were unvaccinated, which reflects statistics in the Bronx during this timeframe [16]. One vaccinated and 7 unvaccinated patients were admitted for worsening COVID-19.

COVID-19 vaccination status is not part of FDA EUA eligibility criteria. Overall admissions in this cohort were lower and median age was younger compared to patients treated at our medical center in early 2021 [10], likely due to expanded EUA criteria allowing treatment of younger patients with fewer comorbidities. Vaccinated patients were less likely to develop COVID-19 at the time, and younger, vaccinated COVID-19 patients were less likely to present to care for mAb treatment. A low rate of admissions among vaccinated individuals suggests that prioritization of unvaccinated or higher risk vaccinated individuals (e.g., age > 65 with more comorbidities, potentially poor vaccine response, or extended interval since last vaccination) is supported.

Our findings indicate that vaccinated, mAb-treated patients were more likely to be older and have more underlying risk factors for development of severe COVID-19. Despite this, vaccinated patients had a low rate of both all-cause (5%) and COVID-related (1%) admission within 30-days, which is likely due to the well documented effectiveness of vaccines in protecting against severe disease, hospitalization, and death [22–27]. Data indicate that most hospitalized COVID-19 patients were unvaccinated during the study timeframe [28–30]. A study of fully vaccinated mAb-treated patients reported that the number needed to treat (NNT) to prevent one hospitalization was 225 among the lowest-risk patients compared to a NNT of 4 among the highest risk patients [31]. Our results also suggest that a “watch and wait” approach is appropriate for fully vaccinated patients with fewer comorbidities versus an immediate test and treat approach for the highest risk patients.

Median duration between vaccination and mAb treatment was high in both admitted and non-admitted patients at 154 and 161 days, respectively, suggesting that waning vaccination-induced antibody levels may have contributed to breakthrough infections in our cohort.

Overall, 30-day admission for mAb untreated, SARS-CoV-2 positive patients at our hospital (regardless of vaccine status) was low at 4% (22/538) between July and August 2021, perhaps due to a high background rate of prior infection (seroprevalence of 24.3% prior to Omicron surge) along with steadily increasing uptake of vaccination [32]. The overall admission rate of mAb-treated patients was similarly low at 6%. Lieberman-Cribbin et al. reported that the Bronx residents are more likely to have increased SARS-CoV-2 seropositivity suggestive of past infection [32].

Acharya et al. demonstrated that there is no significant difference in viral load between vaccinated and unvaccinated patients infected with the SARS-CoV-2 Delta variant [33]. We also did not observe any significant difference in available Ct values between vaccinated and unvaccinated patients, although few vaccinated patients had documented Ct values since most were tested outside of our system. A limitation of our study is that the sample size was only 250 patients with full 30-day follow up, and other mAb-treatment outcomes such as reduction in symptom severity or duration, or spread to secondary contacts, were not evaluated. Additionally, the observational, non-randomized, design limited the ability to draw further conclusions about vaccinated vs. unvaccinated mAb-treatment outcomes. Although morbidity and mortality were high during the Delta surge, the unprecedented Omicron surges have intensified the resource scarcity of mAbs and the need to prioritize patients at highest risk of hospitalization and death. Institutional prioritization criteria utilized during the Delta wave (Table 1) were tightened significantly in December 2021 through February 2022 during the Omicron surge [34].

## Conclusion

Since initial authorization of SARS-CoV-2 mAb therapies, multiple factors have impacted the effectiveness of mAbs, including local access to care and SARS-CoV-2 variants. The possibility of resource scarcity compels infusion programs to prioritize patients at highest risk of poor outcomes from COVID-19. The role of COVID-19 mAb therapies will continue to evolve along with our understanding of the behavior of mAbs in vaccinated vs. unvaccinated hosts and the severely immune compromised.

## Data Availability

The datasets generated and/or analyzed during the current study are not publicly available due to patient privacy and confidentiality but are available from the corresponding author on reasonable request.
